# High Expression of Cyclin D1 and p21 in N-Nitroso-N-Methylurea-Induced Breast Cancer in Wistar Albino Female Rats

**Published:** 2012-12-12

**Authors:** Mahboobeh Ashrafi, Seyedeh Zahra Bathaie, Saeid Abroun

**Affiliations:** 1. Department of Clinical Biochemistry, Faculty of Medical Sciences, Tarbiat Modares University (TUM), Tehran, Iran; 2. Department of Hematology, Faculty of Medical Sciences, Tarbiat Modares University, Tehran, Iran

**Keywords:** Breast Cancer, N-Nitroso-N-Methylurea (NMU), Cyclin D1, p21 Expression

## Abstract

**Objective:**

N-nitroso-N-methylurea (NMU) induces breast cancer in rodents, particularly in rats. This model of breast cancer is very similar to human breast cancer. As a continuation of our recent work, we investigated the expressions of cyclin D1 and p21 in NMU-induced breast cancer of Wistar Albino rats.

**Materials and Methods:**

In this experimental study, mammary carcinoma was induced in female Wistar Albino rats by a new protocol which included the intraperitoneal injection of NMU (50 mg/kg) at 50, 65, and 80 days of the animal’s age. The animals were weighed weekly and palpated in order to record the numbers, location, and size of tumors. Subsequently tumor incidence (TI), latency period (LP), and tumor multiplicity (TM) were reported. About four weeks after the tumor size reached 1.5 cm3, rats were sacrificed. Cyclin D1 and p21 expressions in tumors and normal mammary glands from normal rats were measured by reverse-transcription polymerase chain reaction (RT- PCR) and Western blot analysis. Statistical analysis of the data was performed using SPSS software version 16.0.

**Results:**

The efficiency of tumor induction was 65%, LP was 150 days, and a TM of 1.43 ± 0.53 per rat was noted. RT-PCR and Western blot data indicated significant (p<0.05) induction of both cyclin D1 and p21 expressions in rat mammary tumors compared with normal tissue from the control group.

**Conclusion:**

These results indicate an efficient mammary tumor induction protocol for this type of rat, which is accompanied by an increase in cyclin D1 and p21 expressions.

## Introduction

Breast cancer is the second leading cause of cancer death and the most common malignancy among women (1, 2). About 70% of breast cancers are estrogen-dependent; however, its etiology remains obscure and primary prevention strategies are yet not available. Advances in therapy are limited and alternatives need to be developed for breast cancer control (3, 4).

It has been shown that cell cycle control is an obligatory and conclusive event in tumor development.

Progression in the knowledge about the molecular mechanisms involved in the mammalian cell cycle improves understanding about premalignant lesions, diagnostics, and possible therapeutic protocols (5). The G1 to S and the G2 to M transitions are important points in the control of the cell cycle proliferation of eukaryotic cells, especially in mammalian cells. Cyclins, cyclin-dependent kinases (CDKs), and cyclin-dependent kinase inhibitors (CDKIs) have an important role in these processes (6).

D-type cyclins (D1, D2, D3) have an important role in regulating the G1 checkpoint. D cyclins contribute to the progress of the G1 phase by regulating the activity of CDK4 and CDK6 (7). Cyclin D1 plays a critical role in the development of mammary glands. Its over expression has been reported in 40-90% of cases of invasive breast cancer and at the earliest stages of ductal carcinoma. It is maintained in all stages of metastasis (7, 8).In addition, up-regulation of cyclin D1 expression has been found in hyperplastic mammary glands and proliferative human breast disease. These observations suggest that cyclin D1 may be involved as an important downstream target of diverse upstream signals in mammary gland development and tumorigenesis (9).

On the other hand, the kinase activities of the cyclin/CDK holoenzyme are negatively regulated by CDKIs. Two classes of these compounds are the Cip/Kip and INK4 families (10). Cip/Kip inhibitors can bind to and inhibit both cyclin-D-CDK4/6 kinases, as well as cyclin-E/A-CDK2. The archetypal mammalian CKI and Cip/Kip family is p21 (11).

The etiology of cancer is very diverse. One of the most important factors is chemical carcinogenesis; among which polycyclic hydrocarbons, aromatic amines, and nitrosamines are the most carcinogenetic. Nitrosamines and amides are found in foods such as smoked meat, soused meat, salami, sausage, fish meat, cheese ,and soy oil, in addition to cigarette smoke. Nitrosamines are formed by the combination of nitrogen oxide (NO) originating from nitrate (NO_3_) and nitrite (NO_2_) added with the secondary and tertiary amines formed by the destruction of proteins and amino acids in the gastrointestinal tract (12).

The induction of rodent mammary tumors following the administration of N-nitroso-N-methylurea (NMU) is a widely used experimental model for investigating breast cancer in women. These carcinogen-induced tumors arise from terminal end buds, an analogous structure to the terminal ductal lobular unit in humans, which is the proposed site of the origin of ductal carcinoma in situ (DCIS). Substantial evidence suggests that this animal model mimics human breast cancer (13).The similarities of this tumor with human ER + breast tumors include similarities in histopathology, site of origin, and response to various hormonal manipulations (e.g.ovariectomy, tamoxifen or pregnancy) (14). This model has been used extensively to evaluate the preventative and therapeutic effect of different agents for human breast cancer (13).

Similar to humans, different strains of rats vary considerably in their susceptibility to the development of mammary cancer. Inbred Buffalo (BUF), Wistar-Furth, and inbred and out-bred Sprague-Dawley rats are highly susceptible to multiple mammary carcinomas, even after a single dose of carcinogen treatment. Fischer 344, ACI, and August rats are in the second order of susceptibility and develop less than one carcinoma per animal on average, with a relatively long latency period (LP). On the other hand, inbred Copenhagen (COP) and Wistar-Kyoto rats are extremely resistant. Variability in mammary tumor incidence (TI) rates in the same strain of rat has also been reported by different laboratories. Significant variations, ranging from 10-85%, in the incidence of spontaneously occurring mammary tumors in female rats has been reported (15, 16). However, according to our literature search, there is no efficient method of cancer induction in Wistar Albino rats using NMU. Therefore, in the past few years, we have attempted to design a protocol to induce breast cancer in this strain of rat, that has a high tumor induction efficiency, a reasonable LP, and tumor multiplicity (TM). The expression of cyclin D1 and p21 in these tumors is also investigated as the mechanism involved in carcinogenesis.

## Materials and Methods

### Animals

In this experimental study, female Wistar Albinorats (n=110), 35 days old, were purchased from the Animal Center at Pasteur Institute, Tehran, Iran. They were housed at five animals per cage in a room with controlled lighting (lights on from 06:00–20:00) and temperature of 23 ± 2℃ in the Animal House at Tarbiat Modares University. The animals were fed a standard laboratory diet with access to water ad libitum. They were acclimated for about two weeks before the start of the study. The experimental protocol was approved by the Animal Ethical Committee in accordance with the Guidelines for the Care and Use of Laboratory Animals prepared by Tarbiat Modares University.

We examined different protocols for the induction of breast cancer, that included different doses (50-150 mg/kg), numbers of injections(single or multiple), and time intervals (7, 10, or 15 days)between each treatment (17). From these, we chose the most efficient method to use. NMU was dissolved freshly in 0.9% NaCl and adjusted to pH= 4.0 with acetic acid for activation. Then, we injected NMU (50 mg/kg; Sigma, St. Louis, MO) intraperitoneally into 100 rats, for three times, at 50, 65, and 80 days of age. Animals in control group (n=10) only received vehicle injections. The animals were weighed weekly and palpated in order to record the number, location, and size of tumors.

The following tumor growth parameters were determined: LP, as the number of days between the first NMU injection and the appearance of the first tumor; TI as the percentage of rats that developed at least one tumor; and mean tumor number per rat (n/t), which we defined as the number of tumors per rat in animals that developed at least one tumor.

### Tissue sample preparation

At the end of the study the animals were sacrificed under anesthesia and samples from normal breast or mammary tumors as well as other tissues were quickly removed, weighed, frozen in liquid nitrogen, and stored at -70℃ until use .For histological analysis, the tumor tissues were immediately fixed in 10% formalin, embedded in paraffin wax, and stained with hematoxylin and eosin (H&E).

**Table 1:** Experimental conditions for PCR, including primer sequence, annealing temperature, and expected size of PCR products


Gene	Primer sequence	Product size	Annealing temperature (℃)

Cyclin D1	F: 5'-CAGACCAGCCTAACAGATTTC-3'	208	56
	R: 5'-TGACCCACAGCAGAAGAAG-3'		
P21	F: 5'-CTGGATGCTAGAGGTCTGC-3'	105	58
	R: 5'-AGAGTTGTCAGTGTAGATGC-3'		
GAPDH	F: 5'-CAAGGTCATCCATGACAACTTTG-3'	500.200	58
	R: 5'-GTCCACCACCCTGTTGCTGTAG-3'		
	F: 5'-AACGACCCCTTCATTGAC-3'		
	R: 5'-TCCACGACATACTCAGCAC-3'		


### Reverse-transcription polymerase chain reaction

Total RNA from excised rat mammary glands and tumors was isolated using the TRIzol extraction reagent (Invitrogen, CA, USA), according to the manufacturer’s recommendations. The integrity of mRNA was confirmed by electrophoresis in a denaturing 1% agarose gel and Thermo Scientific Nanodrop 2000C Spectrophotometer. cDNA was synthesized by the RevertAidTMH Minus First Strand cDNA synthesis Kit (Fermentas, Inc., USA) perthe manufacturer’s instructions. A polymerase chain reaction (PCR) was carried out by amplification of genes together with the reference gene (GAPDH) using the cDNA master template, PCR mix (Fermentas, Inc., USA), and specific primers in a MJ Mini™ Personal Thermal Cycler (BioRad, USA). PCR conditions for cyclin D1 amplification were: 30 cycles of 95℃ for 30 seconds, 56℃ annealing for 45 seconds and a 72℃ extension for 45 seconds. PCR conditions for p21 were: 30 cycles of 95℃ for 30 seconds, 58℃ annealing for 45 seconds and a 72℃ extension for 45 second.The primer sequences, product size, and annealing temperature are described in table 1. The primer sequences of GADPH were taken from the cDNA synthesis kit and the literature (18). Cyclin D1 and p21 primers were designed using Oligo 6, Generunner, and Allele ID 07 software. Reaction products were then separated on 2% agarose gel and visualized by ethidium bromide staining. The bands were quantified by densitometric analysis through an image capturing system software. The relative target mRNA expression level was normalized by GAPDH in the same sample.

### Western blot analysis

For Western blotting, frozen tumor and normal mammary gland tissues (about 100 mg) were homogenized in lysis buffer that contained 150 mM NaCl, 50 mM EDTA, 1mM NaF, 10 mM Na4 P2SO7, 0.1% SDS, 100 mM tris-HCl, 1% glycerol, and 1% triton X-100, and a cocktail of protease and phophatase inhibitors (Sigma Chemical Co, USA). Equivalent amounts of protein were applied to 12% SDS-polyacrylamide gels, separated by electrophoresis, and electrotransferred to 0.45 µm pore size polyvinylidene difluoride membranes (Roche, Germany). Membranes were immersed in blocking solution (5% non-fat dry milk, 0.05% Tween 20 in phosphate buffer saline) and incubated overnight at 4℃. Primary incubation of the membranes was carried out by using dilutions of mouse monoclonal anti-p21 (1:100) and anticyclin D1 (1:200) antibodies (#450 and #271610,Santa Cruz Biotechnology, Inc.) for 2 hours at room temperature in 3% milk. After washing, filters were incubated for 1 hour at room temperature with horseradish peroxidase conjugated secondary antibody (1:7000; #2005, Santa Cruz Biotechnology, Inc.). Protein bands were visualized using an ECL Advance Western Blotting Detection Kit (GE Healthcare, Amersham). Equal loading of proteins was assessed by a monoclonal anti-b-actin antibody (#81178, Santa Cruz Biotechnology, Inc).

### Statistical analysis

We analyzed the differences between the data obtained in the control group and the animals with mammary tumors by the independent-samples t test using SPSS version 16.0. P<0.05 was considered statistically significant.

## Results

### General parameters

**Fig 1:**
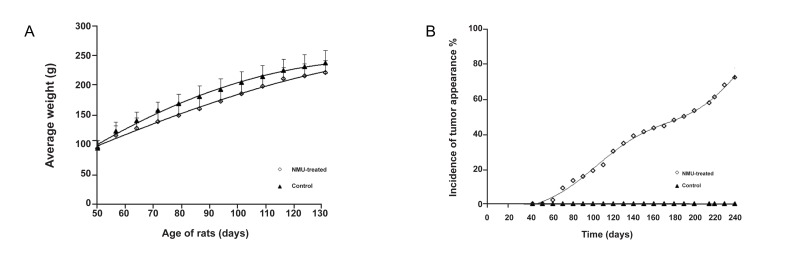
Effects of NMU on rats. A. Changes in the body weight of rats during the course of the experiment. B.The incidence of tumor appearance after NMU injection versus time.

Figure1A shows the changes in weight of all animals during the experiment as well as the lower weight of animals in the NMU-treated group.

In figure1B the percentage of rats with TI is shown. There were no palpable tumors in the control rats.

The first palpable tumor in the NMU-treated group was seen 60 days after the first MNU injection.

 At the end of the study, the average number of tumors per rat in NMU-induced breast cancer was 1.43 ± 0.53. The tumor burden was 7.20 ± 0.85g (total tumor weight per rat) and 3.43 ± 0.76 g (average tumor weight per rat).

 The median tumor time (time needed for the development of tumor in 50% of the animals) was 138 days.Other tumor parameters are recorded in table 2.

**Table 2:** Tumor parameters in NMU-induced breast cancer in rats


Tumor multiplicity (TM)^a^	Tumor latency^b^ (days)	Tumor incidence (TI) (%)^c^	Tumor pathology (%)^d^

1.43 ± 0.53	150.26 ± 56.58	65	91.9


a; Tumor multiplicity ™: Average number of tumors/rat. b; Tumor latency: Lag time between NMU injection and tumor development. c; Tumor incidence (TI): Percent of tumor-positive rats at days post-NMU injection. d; Tumor pathology:Percent of rats with cancerous tumors.

### Histopathology of mammary tumors

**Fig 2:**
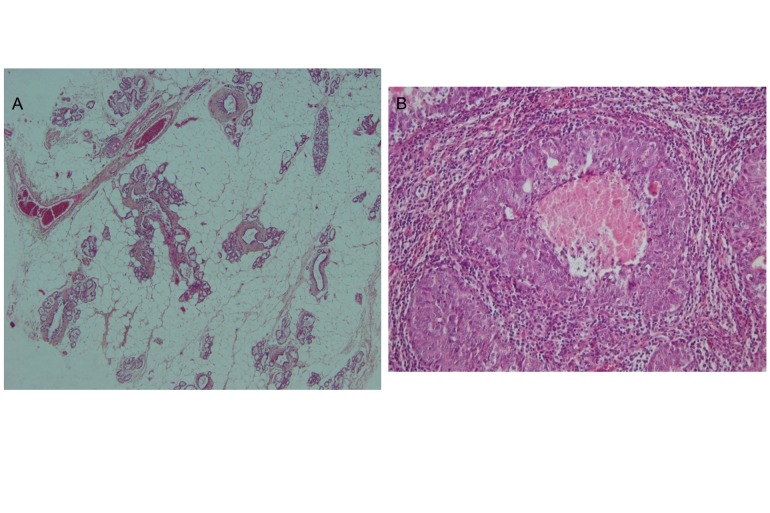
Histopathology of normal tissues (A) and tumor (B). H & E staining of a tumor shows invasive intraductal carcinoma.

H&E stained sections of the samples from this study were analyzed by a specialist in the field of histopathology and are shown in figure 2. The results indicated that 91.9% of tumors from the NMU-treated groups consisted ofdifferent types of malignant adenocarcinoma, such as papillary and comedo carcinoma. A few (8.1%) benign epithelial neoplasms, such as lactating adenoma (adenomas with milk-like substance in the lumen) and papillary adenoma, were observed.

### Cyclin D1 and p21 expression

**Fig 3:**
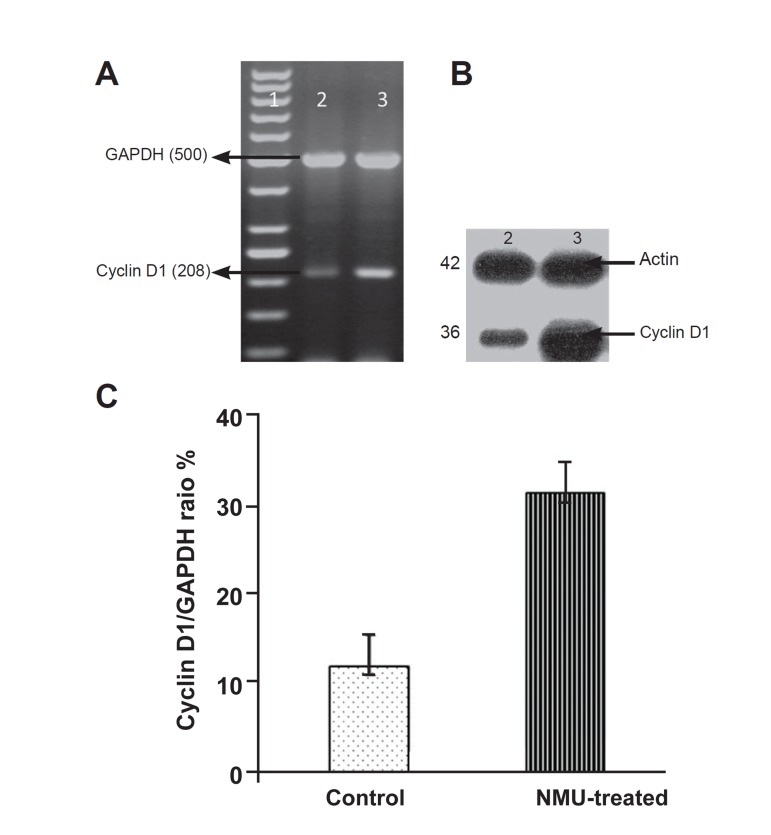
Expression of cyclin D1 at the mRNA level by RT-PCR .A. and protein level by Western blot .B. in rat tumor and normal mammary glands. Lane 1: DNA ladder, lane 2: control (normal mammary gland), lane 3: tumor tissue. C. Densitometric analysis of cyclin D1 expression; values are expressed as mean ± SE, with (p<0.05).

**Fig 4:**
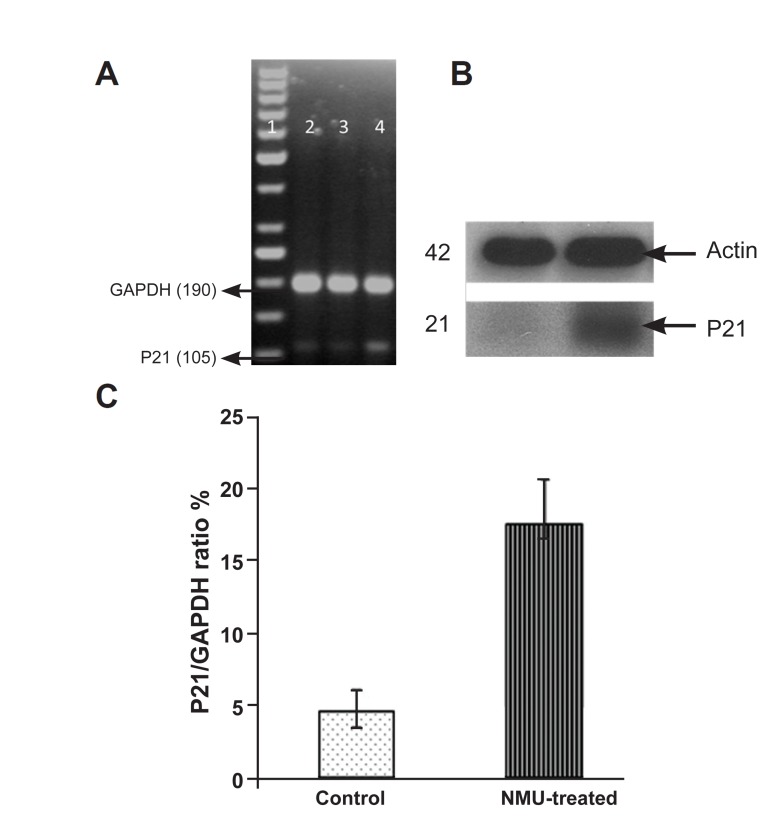
Expression of p21 at the mRNA level by RT-PCR A: and protein level by Western blot. B: In rat tumor and normal ma, mmary gland. Lane 1: DNA ladder, lanes 2, 3: control (normal mammary gland), lane 4: tumor tissue. C: Densitometric analysis of p21 expression; values are expressed as mean ± SE, with (p<0.05)

Cyclin D1 and p21 expression was examined in the mammary tissue of normal rats and in the tumors of NMU-treated rats. RT-PCR and Western blot analysis were performed to determine the mRNA and protein levels of cyclin D1 and CDKIp21 in tumors and normal mammary glands. As seen in the Figures 3 and 4, there is an increase in the mRNA and protein levels of both cyclinD1 and p21 (Cip1) when compared to normal tissue.

## Discussion

Substantial evidence suggests that the induction of mammary carcinomas by NMU in female rats mimics breast cancer in humans. The rat tumor’s histopathology indicates their origination from mammary ductal epithelial cells and their dependency on ovarian hormones for tumor development (ER+) all correlate with human breast cancer (13). Thus this animal model is one of the most frequently used models for investigating breast cancer and mammary tumor treatment (19-21). In contrast to mouse lesions, which are primarily alveolar, both rat and human mammary tumors are predominantly ductal. The most highly malignant rat tumors also share some features with human intraductal and infiltrating ductal carcinomas (20, 21).

It was reported that the NMU model has several advantages, such as TI reliability, organ site specificity, tumor of ductal origin, and predominance of carcinoma, in addition to the ability to examine tumor initiation and promotion processes (22). Generally NMU-induced mammary carcinomas are aggressive and locally invasive with no metastasis.

Comparative studies have also shown that chemically-induced mammary carcinomas, as with their human counterparts, have altered TGFB, erbB2, and cyclin D1 expression (23). Furthermore, some human and most rat mammary tumors also express estrogen and progesterone receptors (24, 25).

We recently examined the induction of breast cancer in rats and the therapeutic effect of some natural compounds on the cancer (17, 26). Our method of tumor induction has been compared with previous methods from other studies that used various races of rats, as shown in table 3 (27-47). The Wistar Albino rat predominantly used in our lab has a median level of suseptibility to cancer. We have analyzed different protocols to induce a stable breast cancer tumor in this rat (17), among which the presented method here was the best choice. Our results showed that with three injections of 50 mg/kg of NMU with a 15 day interval that began from 50 days of the rat’s age, adenocarcinoma was induced in about 91.9% of animals. As shown in table 3, the LP of tumor induction in different races of rats (with an average age of first injection of about 50 days) was between 60-270 days. The obtained LP in our experiment of 60 to 240 days, with an average of 150 days, was acceptable. The observed TI and TM, lack of metastasis and no animal death were the other preferences of this protocol.

It has been reported that rats from other races, such as F344, when administered NMU, develop tumors of a lymphoid origin. Tumors originating from the mammary gland, kidney, muscle, and connective tissue have also been detected (48). In our study there were no tumorsthat had a lymphoid origin. The histopathologic characteristics of mammary tumors in this study showed homogenous tumors that were invasive ductal carcinoma. This result, in addition to the ER (+) character of the NMU-induced mammary tumors, has enabled this model to be suitable for studying the effect of natural products and drugs as preventive or therapeutic agents and to evaluate their efficacy before administration on humans.

Cellular proliferation is regulated by essential checkpoint proteins. Abnormalities in the quantity or activity of these proteins may facilitate uncontrolled cellular proliferation-a commonly reported property associated with carcinogenesis. A positive regulator of the cell cycle is cyclin D1(6).Many oncogenic pathways can up-regulate cyclin D1 in addition to other proliferative, anti-apoptotic, metastatic, and/or angiogenic proteins (1).

The activity of other positive regulators of the cell cycle (CDKs) can be negatively modified by p21 (waf 1, cip 1), which binds to CDKs, causing their inhibition (49). All tumors in this study demonstrated increased expressions of cyclin D1 and p21 compared to normal mammary glands. The increasing expression of p21 with cancer is consistent with the theory that abnormal cells increase p21 in an attempt to 'brake' the process of cellular proliferation at the G1 checkpoint (49).

Over expression of cyclin D1 in NMU-induced breast cancer in rats was shown by Sgambato et al. byWestern blotting (50).The increased expression of this gene and p21 were also reported by Jang et al. (51) in DMBA-induced breast cancer in the Sprague Dawley strain. According to our literature review, the expression of these genes in NMU-induced breast cancer in Wistar albino rats has not been explained previously, and it is the first time that the over expression of both genes and detection of the resulted proteins have been reported.

**Table 3:** Comparison between parameters determined after NMU-induced breast cancer in various races of rats tothe current study


Row	Race	NMU dosage (mg/kg) and injection type	Age at first injection (days)	No. of injections	Time between injections (days)	LP	TI	TM	Histology results	References, Year

1	Sprague-Dawley	50, IP	50	3	30	82	88.4%, metastasis to liver, spleen, and lung	4.4	83.4% carcinoma	30.1998
2	Sprague Dawley	37.5/50, IP	50	1	-	60	55% /75.9%	1.08 ± 0.22 / 2.0 ± 0.35	Carcinoma, papilloma, hyperplasia, carcinoma-in situ	46.1998
3	Sprague Dawley	50, IP	50	2	7	-	-	-	-	38.2000
4	Sprague-Dawley	50, IP	55	1	-	-	-	-	-	32.2000
5	Sprague Dawley	50, IP	50	1	-	63.70	55%	1.3	Adenocarcinoma	43.2001
6	Virgin Wistar	50, IP	50	3	30	113.0 ± 4.2	60%	-	-	28.2004
7	Wistar	50, IV	50-55	3	28	-	-	-	-	31.2004
8	Wistar-furth	50, IP	52	1	-	140	77%	-	Adenocarcinoma	37.2004
9	Virgin wistar	50, IP	50	3	30	113 ± 4.2	60%	1.93 ± 0.4	-	27.41.2005.2008
10	Virgin Sprague Dawley	50,IP	50	1	-	-	93.3%	4.0	-	44.2005
11	Sprague Dawley	50, tail vein injection	50	1	-	78 ± 0.9	83.3%	4.87 ± 0.77	Ductal carcinoma in situ	42.2005
12	Sprague Dawley	50, IP	50	3	50.38	100	-	2 ± 0.63	-	36.2005
13	Sprague Dawley	50, IP	50	1	-	63-70	55%	1.3	Adenocarcinoma	33.2005
14	Lewis, Fischer 344, Wistarfurth, Copenhagen	50, IP	49	1	-	Le: 91, F: 168 W: 252, C: 355	-	-	-	29.2006
15	Sprague Dawley	40,tail vein injection	45	1	-	59.5	100% with 5% metastasis	4.65	Adenocarcinoma, fibroadenoma and tubular adenoma	34.2006
16	Wistar	50	45	2	7	90-120	45%	0.5	Adenocarcinoma	45.2007
17	Sprague Dawley	50, IP	42	2	8	103.09 ± 7.32	55.00% ± 11.41	1.64 ± 0.39	-	40.2007
18	Wistar-furth	50/35, IP	50	50	-	80 ± 2.39	-	-	Ductal carcinoma in situ (DCIS	39.2007
19	Virgin Wistar	50, IP	50	3	7	Up to 210	71%	1.6	Carcinoma	4.2008
20	Copenhagen	50, IP	50-55	1	-	90-270	5%	-	Adenocarcinoma	47.2008
21	Sprague Dawley	50, IP	21	1	-	35	-	4.8 ± 0.6	Adenocarcinomas (papillary, cribriform,	35.2009
22	Sprague Dawley	50, IP	50	1	-	80	80%	2.5 ± 0.34	Adenocarcinoma,benign tumors	1.2009
23	Wistar albino	50, IP	50	3	14	150 ± 56.58	65% without metastasis	1.43 ± 0.53	91.9% adenocar cinoma	Current study


## Conclusion

Breast cancer was induced in female Wistar Albino rats using a novel cancer induction protocol, including three injections of 50 mg/kg NMU beginning from 50 days of the rat’s age and continued with a 15 day interval. Cyclin D1 and p21 expressions were significantly increased in tumor tissues compared with normal mammary gland tissues.
